# Differences in immune-related gene expressions and tumor-infiltrating lymphocytes according to chemotherapeutic response in ovarian high-grade serous carcinoma

**DOI:** 10.1186/s13048-020-00667-y

**Published:** 2020-06-08

**Authors:** Kyung Un Choi, Ahrong Kim, Jee Yeon Kim, Ki Hyung Kim, Chungsu Hwang, So Jung Lee, Won Young Park, Sejin Jung, Hye Jeong Choi, Kyungbin Kim

**Affiliations:** 1 Department of Pathology, Pusan National University Hospital, 179 Gudeok-ro, Seo-gu, Busan, 49241 Republic of Korea; 2 Department of Pathology, School of Medicine, Pusan National University, 49 Busandaehak-ro, Mulguem-eup, Yangsan-si, Gyeongsangnam-do 50612 Republic of Korea; 3 Department of Pathology, Pusan National University Yangsan Hospital, 20 Geumo-ro, Mulguem-eup, Yangsan-si, Gyeongsangnam-do 50612 Republic of Korea; 4 Department of Obstetrics and Gynecology, Pusan National University Hospital, 179 Gudeok-ro, Seo-gu, Busan, 49241 Republic of Korea; 5 Diagnostic Pathology Center, Busan-Gyeongnam Reference Lab., Seegene Medical Foundation, 297 Jungang-daero, Dong-gu, Busan, 48792 Republic of Korea; 6 Department of Pathology, Ulsan University Hospital, 877 Bangeojinsunhwando-ro, Dong-gu, Ulsan, 44033 Republic of Korea

**Keywords:** Ovarian cancer, Immune-related gene, Tumor-infiltrating lymphocyte, Chemotherapeutic response

## Abstract

**Background:**

High-grade serous carcinoma (HGSC) of the ovary is the most common subtype of epithelial ovarian cancer (EOC) and has an overall poor prognosis. There is increasing awareness of the importance of immune cell populations and tumor-infiltrating lymphocytes (TILs) in various immune pathways in the tumor microenvironment. The present study evaluated immune-related gene expressions and TIL levels, as well as associated chemotherapeutic responses, to elucidate the correlation between gene expression and TIL levels in HGSC.

**Materials and methods:**

Fresh tissue samples from 12 HGSC patients were included in this study. Depending on their response to adjuvant chemotherapy, the patients were divided into two groups: chemosensitive (CS) or chemoresistant (CR). The expression levels of 770 genes were analyzed using the nCounter® PanCancer Immune Profiling Panel of the NanoString nCounter® Analysis System. Quantitative real-time polymerase chain reaction (qPCR) was performed to validate the NanoString data obtained. The TIL levels in representative sections were examined via hematoxylin and eosin staining. Gene and TIL levels were subsequently correlated with the chemotherapeutic response.

**Results:**

Several genes were differentially expressed in the two study groups. Eleven representative genes were selected for further evaluation. Of those, 9 genes (IRF1, CXCL9, LTB, CCL5, IL-8, GZMA, PSMB9, CD38, and VCAM1) were significantly overexpressed in the CS group; whereas expressions of 2 genes (CD24 and CD164) were increased in the CR group. Results of qPCR were consistent with those of the NanoString nCounter® analysis. Stromal TIL levels were significantly associated with adjuvant chemotherapeutic response (*p* = 0.001).

**Conclusions:**

Significant differences between the CS and CR groups were observed in the expression levels of immune-related genes. Immune-related gene expressions were significantly higher in the CS group, which also had higher levels of TILs. We, therefore, suggest that, in patients with HGSC, immune-related gene expressions and TIL levels may be associated with chemotherapeutic sensitivity.

## Introduction

Epithelial ovarian cancer (EOC) is a highly malignant neoplasm having a variable response to adjuvant chemotherapy. The majority of early-stage cancers are asymptomatic, and many EOCs are often diagnosed at an advanced stage in conjunction with regional or distant metastases or peritoneal dissemination [1]. Considering these complications, and despite the availability of standardized therapeutic procedures, EOC has a very high mortality rate among all gynecologic cancers. High-grade serous carcinoma (HGSC) of the ovary is the most common subtype of EOC. It is generally treated with cytoreductive surgery and platinum-based combination chemotherapy [2]. Even though patients may initially respond to treatment, the majority of HGSC patients subsequently develop platinum-resistance with relapse, thereby demonstrating an overall poor prognosis [3]. This unmet medical need has resulted in research to develop new potential therapeutic targets for patients with resistance to platinum chemotherapy and to assess the factors responsible for conferring the disparity between platinum-sensitivity and platinum-resistance.

Many immune cell types in the tumor microenvironment interact with the tumor and are involved in identifying and eliminating tumor cells or in affecting the growth and progression of cancer cells by promoting angiogenesis, inducing immune tolerance, and immunoediting [4–7]. The intensity of immune cell infiltration has also been implicated in platinum chemoresistance [3]. Complex immunological processes are regulated by numerous genes. Considering the above, it is necessary to efficiently define the immunologic activities of cancers, as well as to identify changes in immune cell populations, based on the response to adjuvant immunotherapy or chemotherapy.

Several studies have reported that tumor-infiltrating lymphocytes (TILs) in cancers are related to favorable outcomes as well as to an increased response to neoadjuvant chemotherapy [8–10]. Considering recent developments, TILs are believed to reflect a tumor- specific immune response and to represent a potential marker of the intensity of the immune response to cancer [11].

Analysis of tumor immune-related genes of EOC and TILs in the tumor microenvironment could consequently help identify useful validated markers for the management of EOC and elucidate the network of immune genes associated with recurrence, metastasis, or response to chemotherapy. For gene expression analysis in this study, we used the NanoString nCounter® PanCancer Immune Profiling Panel. It is a novel multiplex gene expression panel designed to quantitate 770 genes from 24 different immune cell types and populations, covering both adaptive and innate immune responses, common checkpoint inhibitors, tumor-specific antigens, and housekeeping genes. This panel, therefore, enables the establishment of immune response profiles in all human cancer types and could be applied to identify tumor-specific immune targets. The NanoString nCounter® Analysis System is a highly sensitive and fully-automated system that allows simultaneous direct measurement of multiplex gene expressions while using a minute amount of total mRNA (25–300 ng) without the use of enzymes or amplification.

The present study aimed to evaluate the expressions of immune-related genes considered potential targets for new treatment strategies, assess the level of TILs following the chemotherapeutic response in EOC, and clinically assess their predictive value or prognostic significance for the chemotherapeutic response.

## Materials and methods

### Patients and tissue samples

Fresh EOC tissue samples were obtained by surgical resection and stored at the National Biobank of Korea, Pusan National University Hospital Cancer Center, Republic of Korea. Twelve patients (age range, 42 to 79 years; mean, 61.2 years) who underwent surgical resection for EOC were examined. The present study was approved by the Institutional Review Board at Pusan National University Hospital. The tumor had been optimally debulked, and all patients received adjuvant chemotherapy involving a paclitaxel and carboplatin-based combination regimen. Various clinicopathological data, such as histologic tumor type, tumor grade, clinical surgical stage, chemotherapeutic regimen, and survival records, were obtained after reviewing medical records and pathology reports of the patients. Surgical staging was based on criteria recommended by the International Federation of Gynecology and Obstetrics (FIGO). Tumor histologic type and grade were determined following World Health Organization (WHO) criteria.

For comparative analyses, patients were divided into two categories: chemosensitive (CS) versus chemoresistant (CR) groups, depending on their response to adjuvant chemotherapy. Tumor response assessment was based on relapse time after the first cycle of adjuvant chemotherapy. Patients with no relapse or recurrence at 1 year or more after the end of their first adjuvant chemotherapy were included in the clinical CS group. The CR group included patients classified as refractory, resistant, or partially resistant: no response or progression during treatment was defined as refractory or intrinsic resistance; recurrence within 6 months of ending chemotherapy was defined as resistant or acquired resistance; recurrence between 6 months and 1 year after completing chemotherapy was defined as partially resistant [2, 12].

Formalin-fixed paraffin-embedded blocks were made for all 12 cases and cut into 4 μm-thick sections for hematoxylin and eosin (H&E) staining.

### NanoString nCounter® PanCancer immune profiling panel for gene expression analysis

The nCounter® PanCancer Immune Profiling Panel (NanoString Technologies, Inc., Seattle, WA, USA) is a unique 770-multiplex gene expression panel that can be used to determine the human immune response in all cancer types [13]. Herein, total RNA was extracted using the RNeasy Mini Prep Kit (Qiagen, Valencia, CA, USA). The RNA yield and purity were assessed using a DS 11 Series Spectrophotometer (Denovix Inc., DE, USA). Total RNA (100 ng) was assayed on the nCounter Digital Analyzer (NanoString Technologies) according to the manufacturer’s protocol. Briefly, hybridizations were carried out by combining 5 μL of each RNA sample with 8 μL of nCounter Reporter probes in the hybridization buffer and 2 μL of the nCounter Capture probes (total reaction volume of 15 μL), and the mixture was incubated overnight at 65 °C for 16–30 h. Excess probes were removed by performing two-step magnetic bead-based purification on the nCounter Prep Station (NanoString Technologies).

Amounts of specific target molecules were quantified on the nCounter Digital Analyzer by counting the individual fluorescent barcodes and assessing the target molecules. For each assay, a high-density scan encompassing 280 fields of view was performed. After obtaining images of the immobilized fluorescent reporters in the sample cartridge with a CCD camera, the data were collected using the nCounter Digital Analyzer. The mRNA data analysis, including the determination of fold change, was performed using nSolver™ software (freely available from NanoString Technologies). The mRNA profiling data were normalized using housekeeping genes. Fold changes greater than 1.8-fold upregulation or 1.8-fold downregulation were considered significant. After initial analysis, genes presenting more than 1.8-fold changes and with *p*-values < 0.05 between the two groups were selected, but such genes were excluded if there were less than 20 negative control counts. The gene expression heat map for genes expressed differently in the CS and CR groups was plotted and analyzed using R software. All processes were performed only once.

### RNA extraction and quantitative real-time polymerase chain reaction (qPCR)

Total RNA was purified from cells using the RNeasy Mini Prep Kit (Qiagen, Valencia, CA, USA). The cDNA was synthesized from 1 μg of RNA by using the ProtoScript® First Strand cDNA Synthesis Kit (New England Biolab Inc., MA, USA). Differential RNA levels were assessed using the Luna Universal qPCR Master Mix (New England Biolab Inc., MA, USA) and the appropriate primer for each gene. Quantitative real-time PCR (qPCR) was performed on an ECO Real-Time PCR system, and the results analyzed using ECO ware (PCRmax, Beacon Road, Staffordshire, UK). The qPCR was carried out three times with the same settings and samples in order to estimate precision and improve experimental variation; the average value was used for evaluation. All samples were normalized to the signal generated from GAPDH using the following primers: forward 5′- GAAGGTGGTGAAGCAGGC and reverse 5′- CTCCTTGGAGGCCATGTG. Primer sequences of each gene are presented in Table 1. The ∆CT is the difference in threshold cycles (CT) between the target and reference genes, and the ∆∆CT is the difference in ∆CT between target and reference samples, which was obtained using the formula ∆∆CT = ∆CT (target sample) − ∆CT (reference sample). For comparisons between the two groups, the relative gene expression reference value was set as 1.

**Table 1:** Forward and reverse primers used for qPCR

Gene	Forward primer	Reverse primer
CCL5	AGTGCTCCAACCCAGCAG	GGGAAGCCTCCCAAGCTA
CD38	GGATGCTTTCAAGGGTGC	GCCTAGCAGCGTGTCCTC
IRF1	AGGCCAACTTTCGCTGTG	GCTGGAATCCCCACATGA
CXCL9	GCCACCGAGATCCTTATCG	CCACATCCTGCAGAGGCT
PSMB9	CTGGGACCAACGTGAAGG	ATGGCCAGAGCAATAGCG
LTB	GGGTTTCAGAAGCTGCCA	CGGTAGCCGACGAGACAG
GZMA	CAGTTGTCGTTTCTCTCCTGC	TGAGCCCCAAGAATGACC
VCAM1	AAAACAATGAGCTGAGAGGCA	TCAAGGAACTCCTCCAGTTCTC
CD24	GCACCCAGCATCCTGCTA	GCCTTGGTGGTGGCATTA
IL-8	TCCATAAGGCACAAACTTTCA	CCTTGGCAAAACTGCACC
CD164	TTGGGGAAAGGTCGGTTT	CATGAATGTGTGTCAGGGAA

### Histological evaluation for tumor-infiltrating lymphocytes

Complete sections of H&E-stained slides were reviewed under a light microscope. A representative tumor section was selected to assess the stromal TIL percentage, which, as recommended by the International TILs Working Group [14], was defined as the mean percentage of the stromal area occupied by mononuclear inflammatory cells over the total intratumoral stromal area. Stromal TILs are defined as mononuclear inflammatory cells located in the stroma between, but not in direct contact with, the tumor cells. TILs in tumor zones with crush artifacts, necrosis, or hyalinization were excluded. Under × 200 magnification, all mononuclear cells were scored, including lymphocytes and plasma cells, but excluding granulocytes and other polymorphonuclear leukocytes. According to the International TILs Working Group’s recommendation [14], one section per patient is currently deemed sufficient for TIL evaluation; regardless, we assessed all available sections from 3 to 8 fields in each patient. Two pathologists evaluated the stromal TILs together and made a consensus-based decision when their evaluations were considerably different. A full assessment of average TIL levels in the tumor area was undertaken, with the assessment not just focused primarily on hotspots. Statistical analysis was conducted to evaluate observed differences in TIL levels of the CS and CR groups.

### Statistical analysis

All statistical analyses were performed using SPSS for Windows software, version 21.0 (IBM Corp., Armonk, NY, USA). Independent sample t-tests and normality tests were used to evaluate the correlation of TIL levels between groups. Fisher’s exact test was applied to assess the correlations between the two groups and their clinicopathological parameters. A *p*-value < 0.05 was considered significant.

## Results

### Clinicopathological characteristics of all 12 patients

The study cohort comprised 6 cases each in the CS and CR groups. All 12 patients were women aged 42 to 79 years (mean, 61.2 years). The mean ages of the CS and CR groups were 58.17 ± 8.08 years and 64.17 ± 12.64 years, respectively (*p* = 0.35). Patient follow-up was done from the date of surgery until either death or last visit to the outpatient department. The follow-up period ranged from 10 to 75 months (mean, 34.8 months). All patients were histologically diagnosed as HGSC and received post-operative adjuvant chemotherapy with the same paclitaxel and carboplatin regimen. Of the total cases analyzed, 1 was FIGO stage II, 7 were FIGO stage III, and 4 were FIGO stage IV. In the CR group, 1 patient was FIGO stage II, 4 were FIGO stage III, and 1 was FIGO stage IV. In the CS group, 3 patients each were FIGO stage III and FIGO stage IV. Patients of both groups showed no statistically significant difference in FIGO stage distribution (*p* = 0.546). The tumors either recurred or progressed in 8 of the 12 patients. The free interval between the end of the initial chemotherapy and recurrence, known as the platinum-free interval, ranged from 0 to 32 months. A platinum-refractory tumor, indicative of progressive disease, was detected in 1 patient during the initial chemotherapy and was considered to exhibit refractory (intrinsic) resistance to platinum-based treatment. During the follow-up period, 4 patients in the CS group experienced no disease relapse. In the CR group, 3 patients died, the status of 2 were unknown, and 1 was alive. All patients of the CS group were alive at the end of the follow-up period. Table 2 presents a summary of the clinicopathological features of the 12 cases.

**Table 2:** Clinicopathological features of epithelial ovarian cancer patients of all 12 cases

Case	Age	FIGO stage	Pathologic diagnosis	CTx regimen	Follow-up period (mo)	Time of recurrence after CTx (mo)	Response group	Status of survival
1	42	IIIc	HGSC	Carbo-Taxol	15	4	CR	No
2	63	IV	HGSC	Carbo-Taxol	29	8	CR	No
3	79	IIIc	HGSC	Carbo-Taxol	17	4	CR	N/A
4	66	IIIc	HGSC	Carbo-Taxol	29	Progression during CTx	CR	N/A
5	62	IV	HGSC	Carbo-Taxol	10	1	CR	No
6	73	IV	HGSC	Carbo-Taxol	30	Relapse within CTx	CR	Yes
7	64	IIIc	HGSC	Carbo-Taxol	35	No relapse	CS	Yes
8	55	IIIc	HGSC	Carbo-Taxol	51	No relapse	CS	Yes
9	62	IIIc	HGSC	Carbo-Taxol	64	No relapse	CS	Yes
10	69	IIa	HGSC	Carbo-Taxol	39	32	CS	Yes
11	50	IIIc	HGSC	Carbo-Taxol	24	No relapse	CS	Yes
12	49	IV	HGSC	Carbo-Taxol	75	19	CS	Yes

*FIGO* the International Federation of Gynecology and Obstetrics, *HGSC* High-grade serous carcinoma, *CTx* chemotherapy, *Carbo-Taxol* carboplatin and paclitaxel, *mo* month, *CR* chemoresistant, *CS* chemosensitive, *N/A* not applicable

### Gene expression differences between the CS and CR groups

**Fig. 1:**
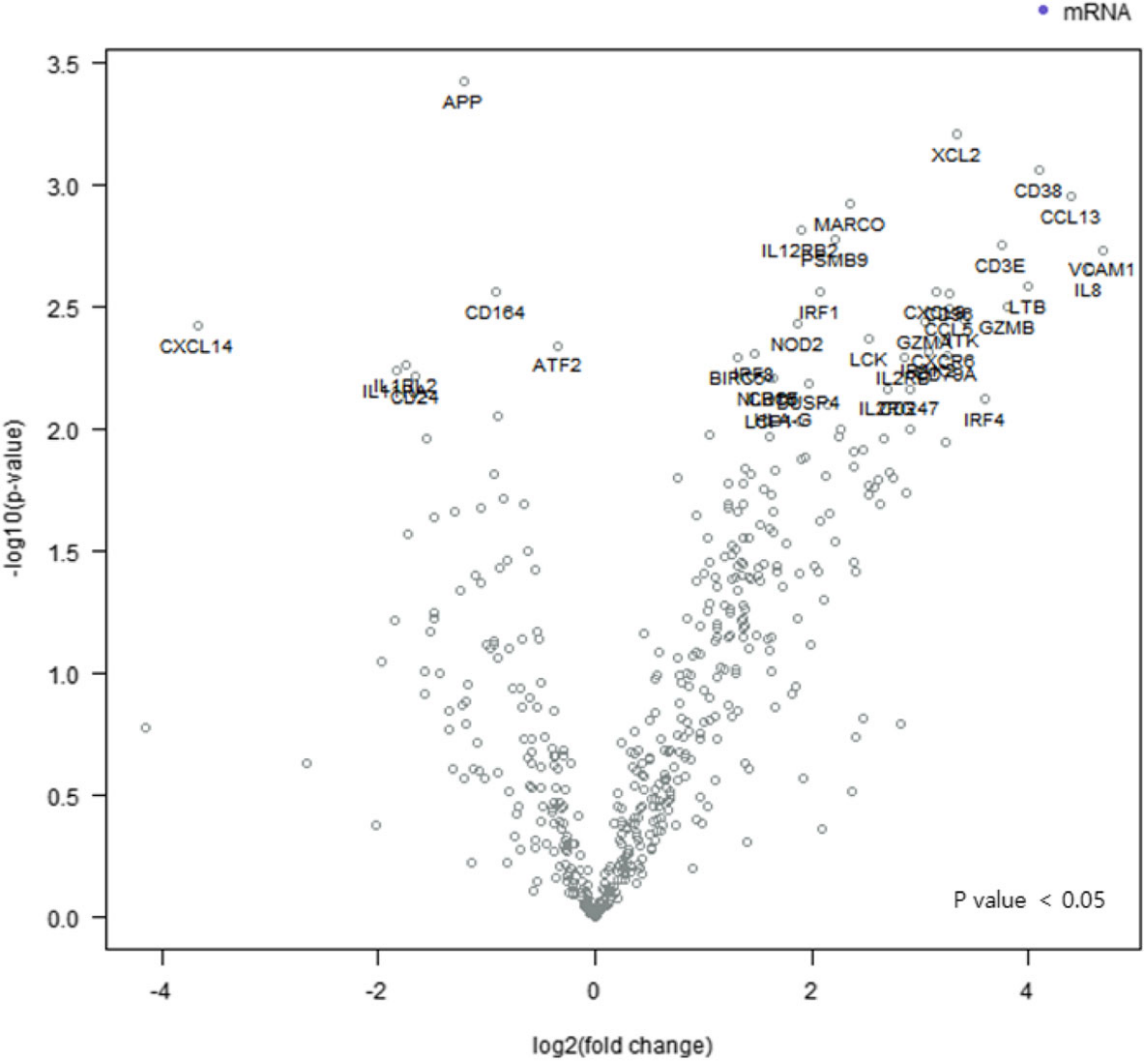
Volcano plot showing differential expression of genes related to the CS group. Data-points (genes) that are located toward the top of the plot and to the right of center are considered to have high statistical significance. Significant immune-related genes related to the CS group, such as IRF1, CXCL9, LTB, CCL5, IL-8, GZMA, PSMB9, CD38, and VCAM1, are identified in the right upper quadrant of the plot, an area that indicates overexpression. (CS: chemosensitive)

**Fig. 2:**
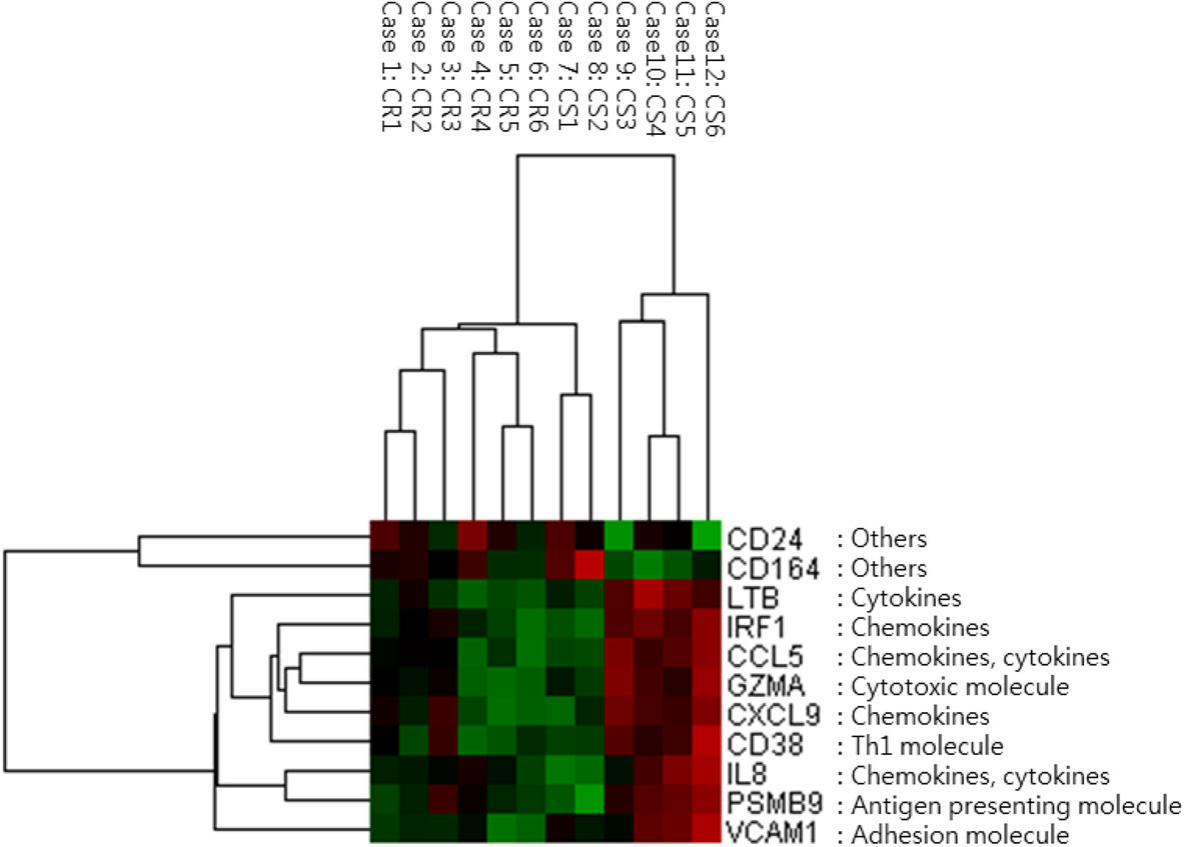
Heat map generated from mRNA data for 11 genes with different expression levels in the CS and CR groups. Color scale: red indicates highly expressed genes. (CS: chemosensitive, CR: chemoresistant)

Gene expressions in both groups were compared to identify genes expressed differently in the two groups. In the 770-multiplex gene panel of the NanoString nCounter® PanCancer Immune Profiling Panel, the significant immune-related genes related to the CS group are presented in Fig. 1. Seventy-two genes were expressed differently in the groups. Sixty-three genes (IRF1, CXCL9, LTB, CCL5, IL-8, GZMA, PSMB9, CD38, VCAM1, TRAF3, CTSL, PIK3CG, IL4R, FCGR2A, CSF3R, IL16, VEGFA, TNFAIP3, CCL3L1, IL32, AMICA1, TP53, CSF2RB, PSMB10, ITGAM, TTK, HCK, PTPRC, BIRC5, FCER1G, CDK1, CD44, CYBB, HLA-DRB3, CCR1, PSMB8, TNF, CD48, ITGAX, JAK3, CCL2, HAVCR2, IL15RA, RIPK2, SLC11A1, TAP2, HLA-A, ISG20, NOD2, CCL4, LAMP3, MICB, FCGR3A, HLA-B, HLA-DMB, LCP1, HLA-G, IRAK2, TAP1, CCL8, IL2RG, CXCL10, and LCN2) and 9 genes (CD24, CD164, CREB5, APP, CYFIP2, JAM3, CX3CR1, TFEB, and ENG) were highly expressed in the CS and CR groups, respectively (Table 3). Based on the obtained gene expression levels and observed fold changes with low *p*-values, 11 candidate genes were selected as potential EOC immunotherapy targets: IRF1, CXCL9, LTB, CCL5, IL-8, GZMA, PSMB9, CD38, VCAM1, CD24, and CD164. For each of those genes, fold changes of the CS group expression compared to the CR group expression were 3.29, 7.07, 6.02, 5.24, 9.08, 4.68, 4.32, 7.73, 6.23, − 3.65, and − 1.8, respectively (Table 4) [15]. The difference among the genes expressed in the CS and CR groups is notably distinct, and 11 candidate gene expressions were significantly different between the two groups. However, the clustering presented by the heat map showed that two of the CS cases (cases 7 and 8) closely resembled CR cases (Fig. 2).

**Table 3:** Total 72 genes expressed differently in the groups by NanoString analysis

	Highly expressed genes
CS group	IRF1, CXCL9, LTB, CCL5, IL-8, GZMA, PSMB9, CD38, VCAM1, TRAF3, CTSL, PIK3CG, IL4R, FCGR2A, CSF3R, IL16, VEGFA, TNFAIP3, CCL3L1, IL32, AMICA1, TP53, CSF2RB, PSMB10, ITGAM, TTK, HCK, PTPRC, BIRC5, FCER1G, CDK1, CD44, CYBB, HLA-DRB3, CCR1, PSMB8, TNF, CD48, ITGAX, JAK3, CCL2, HAVCR2, IL15RA, RIPK2, SLC11A1, TAP2, HLA-A, ISG20, NOD2, CCL4, LAMP3, MICB, FCGR3A, HLA-B, HLA-DMB, LCP1, HLA-G, IRAK2, TAP1, CCL8, IL2RG, CXCL10, and LCN2
CR group	CD24, CD164, CREB5, APP, CYFIP2, JAM3, CX3CR1, TFEB, and ENG

*CS* chemosensitive, *CR* chemoresistant

**Table 4:** Top 11 genes with significant expression by NanoString analysis (the value of the CS group compared to the CR group)

Genes	Fold changes	***P***-value	Gene sets [15]
IRF1	3.29	0.00276	Chemokines, NK cell functions, regulation, T-cell functions
CXCL9	7.07	0.00275	Chemokines, regulation, T-cell functions
LTB	6.02	0.00263	Cytokines, TNF superfamily
CCL5	5.24	0.00322	Chemokines, cytokines
IL-8	9.08	0.00224	Chemokines, cytokines, interleukins, pathogen defense, regulation
GZMA	4.68	0.00364	Cell functions, cytotoxicity
PSMB9	4.32	0.00169	Antigen processing
CD38	7.73	0.00008	B-cell functions, regulation, T-cell functions
VCAM1	6.23	0.00185	Adhesion, regulation
CD24	- 3.65	0.00612	–
CD164	- 1.80	0.00277	–

*CS* chemosensitive, *CR* chemoresistant

The molecules were classified based on the primary function of each gene: chemokines or cytokines (IRF1, CXCL9, LTB, CCL5, and IL-8), cytotoxic molecule (GZMA), antigen-processing molecule (PSMB9), Th1 molecule (CD38), and adhesion molecule (VCAM1). The CD24 and CD164 molecules are placed in other categories. Nine of the 11 candidate genes, namely IRF1, CXCL9, LTB, CCL5, IL-8, GZMA, PSMB9, CD38, and VCAM1, were highly overexpressed and significantly associated with the CS group. Expressions of the CD24 and CD164 genes were considerably decreased in the CS group; the high expression levels of CD24 and CD164 were associated with the CR group.

**Fig. 3:**
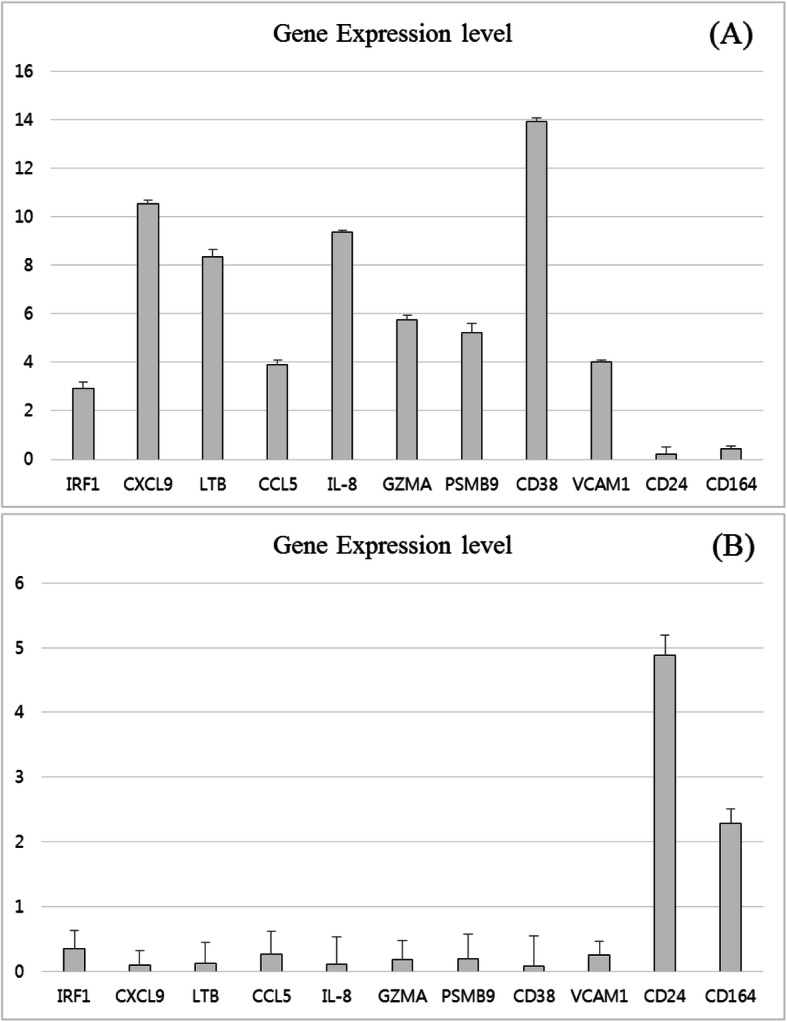
Quantitative real-time PCR validation of NanoString-derived results. The PCR results showed that genes were differentially expressed in the CS and CR groups. Gene expressions of CCL5, CD38, IRF1, CXCL9, PSMB9, LTB, GZMA, VCAM, and IL-8 were considerably high in the CS group (**a**). In contrast, CD24 and CD164 had significantly high expression in the CR group (**b**) (reference value = 1). (CS: chemosensitive, CR: chemoresistant)

To compare and validate the gene expression results obtained via the NanoString method, qPCR was performed. The qPCR results showed that the CS group overexpresses IRF1, CXCL9, LTB, CCL5, IL-8, GZMA, PSMB9, CD38, and VCAM1 mRNA (Fig. 3a), and the ∆∆CT value of each of those genes was − 1.55, − 3.40, − 3.06, − 1.96, − 3.23, − 2.52, − 2.39, − 3.80, and − 2.00, respectively, and their relative values were determined to be 2.94, 10.54, 8.35, 3.88, 9.37, 5.75, 5.24, 13.92, and 4.01, respectively (data not shown). Compared to the CS group, the mRNA expressions of CD24 and CD164 were notably increased in the CR group (Fig. 3b), showing relative values of 4.88 and 2.29, respectively (data not shown). Taken as a whole, the results obtained via qPCR and from the NanoString nCounter® Analysis System were fully concordant.

### Comparison of TIL levels between the CS and CR groups

**Fig. 4:**
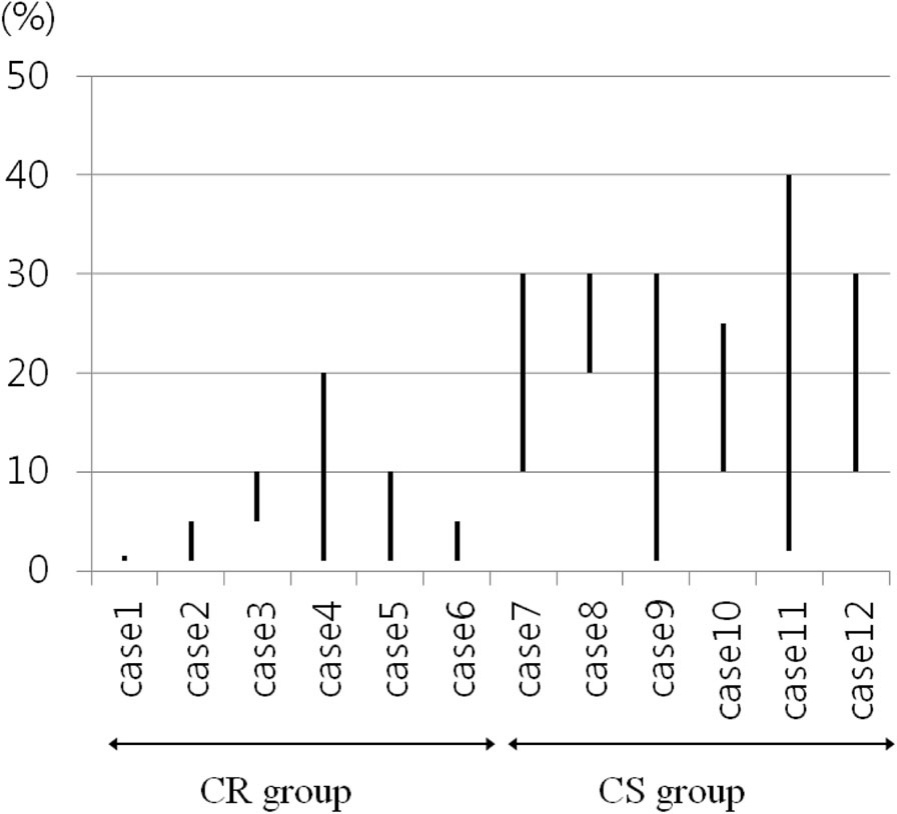
Range of stromal tumor-infiltrating lymphocyte (TIL) levels in all 12 cases. Each case exhibited different levels of TILs as follows: case 1, all 1%; case 2, 1 to 5%; case 3, 5 to 10%; case 4, 1 to 20%; case 5, 1 to 10%; case 6, 1 to 5%; case 7, 10 to 30%; case 8, 20 to 30%; case 9, 1 to 30%; case 10, 10 to 25%; case 11, 2 to 40%; case 12, 10 to 30%. Cases 1 to 6 are included in the CR group, and cases 7 to 12 are included in the CS group. Compared to the CR group, the CS group generally showed higher TIL levels. (CR: chemoresistant, CS: chemosensitive)

**Fig. 5:**
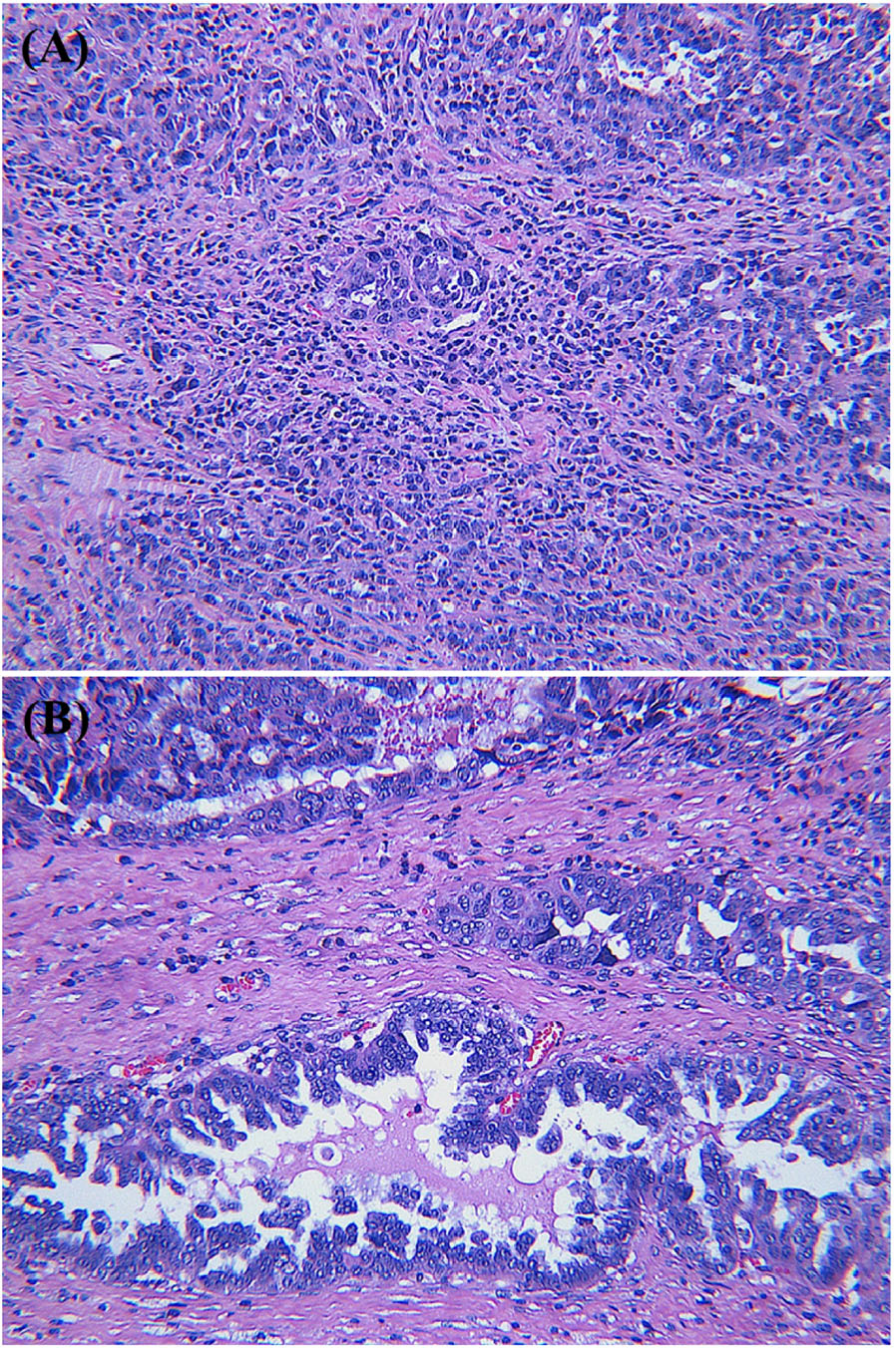
Stromal tumor-infiltrating lymphocytes (TILs) in representative cases of the CS and CR groups. Case 12 of the CS group had abundant TILs with a TIL level of 16.43% (**a**), whereas case 6 of the CR group showed dispersed TILs with a TIL level of 2.33% (**b**). (hematoxylin and eosin stain; magnification, (**a**) × 200, (**b**) × 200). (CS: chemosensitive, CR: chemoresistant)

TILs were investigated to assess the correlation between the TIL level and the chemotherapeutic response. TIL levels were scored by pathologists blinded to the NanoString nCounter® and qPCR results. In addition, the pathologists were blinded to the patients’ chemoresponsiveness. The H&E-stained tissue sections indicated the average percentages of stromal TILs in the CS and CR groups ranged from 8.25 to 27.50% (mean 17.06 ± 2.56%) and 0.83 to 7.33% (mean 4.27 ± 6.30%), respectively (Table 5). The range of TILs recorded for each case in the study is presented in Fig. 4. Higher TIL levels were significantly associated with the CS group, whereas the CR group showed lower levels of TIL (*p* = 0.001). Representative cases within each group exhibiting an abundance of TILs (case 12, CS group) or dispersed TIL infiltration (case 6, CR group) are shown in Fig. 5.

**Table 5:** Stromal TIL levels in all 12 cases

Case	Response group	TIL levels (%)	Mean level of TILs (%)	***P***-value
1	CR	0.83	4.27 ± 6.30	0.001
2	CR	3.20		
3	CR	6.67		
4	CR	7.33		
5	CR	5.25		
6	CR	2.33		
7	CS	14.00	17.06 ± 2.56	
8	CS	27.50		
9	CS	8.25		
10	CS	18.33		
11	CS	17.83		
12	CS	16.43		

*TIL* tumor-infiltrating lymphocyte, *CS* chemosensitive, *CR* chemoresistant

## Discussion

The current study investigated the expression of diverse immune-related genes and TILs in EOCs according to chemotherapeutic response and evaluated the relationships between immune-related gene expressions and TIL levels. Assays were performed on the fully-automated and highly precise NanoString nCounter® Analysis System. That system allows for direct multiplexed measurements of gene expressions in samples with low amounts of mRNA without the need for amplification.

The expressions of immune-related genes in EOCs (especially HGSC) were evaluated, and several genes were shown to have predictive value for the type of adjuvant chemotherapeutic response and prognostic significance in EOC patients. Our results indicate that the CS group includes cases with considerable overexpression of chemokines or cytokines (IRF1, CXCL9, LTB, CCL5, and IL-8), as well as cytotoxic (GZMA), antigen-processing (PSMB9), Th1 (CD38), and adhesion (VCAM1) molecules. Typically, IRF1 (interferon regulatory factor-1) can regulate the transcription of genes related to immunomodulatory and antiproliferative effects [16]. The activation of IRF1 involves the development of CD8+ T cells and natural killer (NK) cells, as well as T-helper cell differentiation [17]. Moreover, it acts as a tumor suppressor and stimulates an immune response against tumor cells. CXCL9 (chemokine ligand 9) mediates the recruitment of tumor-suppressive CXCR3+ T cells and NK cells and is significantly associated with improved prognoses for breast and colorectal cancers [18–20]. Correspondingly, high levels of these chemokines in the tumor microenvironment have been associated with increased numbers of TILs and favorable survivals in renal cell carcinoma and ovarian carcinoma [10, 21]. LTB (lymphotoxin β) is a tumor necrosis factor (TNF)-related cytokine that initiates extrinsic apoptotic cell death in tumor cells via the LTβR signaling pathway [22, 23]. CCL5 (chemokine ligand 5) is chemotactic for T cells, eosinophils, and basophils, and it activates immune cells (including specific NK cells), thereby exerting anti-tumor immunity or immune surveillance; however, several studies have reported that CCL5 is associated with cancer progression [24–26]. GZMA (granzyme A) is the most abundant cytotoxic granule released by CD8+ cytotoxic T lymphocytes and NK cells; it participates in the immune elimination of viruses, bacteria, and tumors by activating unique target-cell death pathways [27]. Narayanan S et al. showed that GZMA-induced high cytolytic activity and perforin expression are related to improved colorectal cancer survival [28]. PSMB9 (proteasome subunit beta type 9) is an immunoproteasome that primarily processes antigens for the presentation of major histocompatibility complex class I molecules to CD8+ T cells [29]. CD38, as a pleiotropic cell surface molecule, interacts with innate and adaptive immune responses via migration, survival, and the T-helper 1 (Th1) polarization ability of dendritic cells [30]. VCAM1 (vascular cell adhesion molecule-1) is an endothelial adhesion receptor having a vital function in immune surveillance by mediating leukocyte transendothelial recruitment [31]. Lastly, IL-8 (interleukin-8) is a proinflammatory chemokine that is associated mainly with the promotion of neutrophil chemotaxis. Interestingly, recent studies have demonstrated that intratumoral IL-8 expression is a crucial regulator of the infiltration of neutrophils into the tumor microenvironment, and in the enhancement of proliferation and survival of cancer cells [32, 33]. Considering our results, we understand that various immune-related genes are directly or indirectly expressed in the CS group and that the immune system response is effective in CS-type EOCs.

Conversely, the CR group exhibits decreased expressions of the 9 genes mentioned above that were highly expressed in the CS group. However, the CD164 and CD24 genes were highly expressed in the CR group patients, not in those of the CS group. The cell surface protein CD24 participates in various processes, including adaptive immune response, autoimmune disease, inflammation, and cancer [34]. Furthermore, recent studies have described CD24 as a putative marker for cancer stem cell (CSC) populations associated with aggressive cancer types and poor prognoses [35–37]. CSCs are important in the initiation of tumors, and they contribute to the incompletion of responses to chemotherapy and radiotherapy [38–40]. Gao et al. revealed that CD24 is detected in ovarian CSCs, and CD24+ cells have a strong resistance to chemotherapy agents and a high self-renewal ability [36]. Kiyoko K et al. indicated that CD24 induces epithelial-mesenchymal transition via both the PI3K/Akt and ERK pathways, and CD24+ cells are related to cisplatin resistance in ovarian cancer cells [41]. The present study also indicates that a high expression of CD24 is associated with an inadequate chemotherapeutic response. This observation may be considered additional evidence for a significant role of CD24 in EOCs. In addition, CD164 (a hematopoietic stem cell surface marker) promotes lung tumor-initiating cells and chemoresistance via Akt/mTOR signaling [42].

Recent data indicate that CSCs promote macrophage polarization toward immunosuppressive phenotype (tumor-associated macrophages, TAMs) and inhibit the expansion of antitumorigenic immune cells, even though a CSC-specific immune interaction is complicated and only focally interpreted [43]. The exact roles of CD24 and CD164 in the immune mechanism of EOC remain unclear; thus, additional studies are required to explain their functions within the immune mechanism. However, since they are associated with stem cell markers, it could be presumed that they impart chemotherapeutic resistance, as has been reported previously. Therefore, investigating CSCs or CSC-specific immunity will be helpful in the development of immunotherapeutic approaches aimed at the elimination of chemotherapy-resistant CSCs.

The current study was designed to allow comparisons between the HGSC patient groups, not between individuals. Thus, the average gene expression levels of each group, rather than individual values, were used in the analyses. As well, the small number of cases (only 12 samples) in this study could be considered a study limitation. Due to the limitations of the present study, it would be necessary to undertake additional studies involving larger sample sizes and other experimental approaches to expand on the results of this study.

It is generally accepted that the presence of TILs, especially intraepithelial cytotoxic CD8+ T cells, is a favorable prognostic factor in EOCs [44–46]. However, the prognostic value of stromal TIL levels in ovarian cancer remains unclear. Some studies have shown a negative correlation or no correlation between stromal TIL level and survival [47, 48]. Other results have indicated the significance of intraepithelial TILs in EOCs, and recent studies have determined that assessment of stromal TILs provides a reproducible and superior prognostic parameter [49], as mentioned by the TILs Working Group [14]. It is, therefore, necessary to assess further the prognostic significance and potentially predictive value of stromal TIL levels in EOCs. To that end, and based on recommendations of the International TILs Working Group, stromal TIL levels were evaluated in this study. The present study revealed that higher average levels of TILs were associated with the CS group. That observation is consistent with the chemotherapeutic sensitivity results and the differences in the expressions of immune-related genes in the two groups. It can be suggested that EOCs, similar to other solid tumors, are immunogenic; therefore, immune cells may play a key role and be potential therapeutic targets in EOCs. Although the composition of the TILs examined in the current study was not analyzed, several other studies have reported that CD3+, CD8+, and CD4+ TILs are associated with good prognoses [45, 50]. Our results showed that a high TIL level corresponded with the increased expressions of immune-related genes in the CS group; therefore, the expression of immune-related genes might be correlated with the TIL level.

In conclusion, gene expression profiling is a potent tool that can provide information useful in the development of predictive biomarkers for application in immunotherapy, personalized medicine, and prognosis prediction in cancer patients. We detected several attractive potential predictive targets using the NanoString nCounter® PanCancer Immune Profiling Panel, a reliable approach to assessing multiple candidate genes simultaneously. Eleven immune-related genes were selected and found to be associated with the TIL level and the type of chemotherapeutic response. Thus, there is a possibility of using those 11 genes as potential markers for predicting the immune or chemotherapeutic response of EOCs or for use in estimating immunotherapeutic susceptibility in EOCs. Notably, we propose that CD24 and CD164 could be used as negative predictors of an adjuvant chemotherapeutic response in patients with EOCs, in particular HGSC, and considered for use in establishing new alternative treatment strategies in patients with chemoresistant EOCs.

## Data Availability

The raw data used and analyzed during the current study are available from the author upon request. The raw data have been deposited in GEO and the accession number to GEO is GSE148392.
